# Microcirculation Monitoring in Septic Shock: Focused Review

**DOI:** 10.3390/medicina62020346

**Published:** 2026-02-09

**Authors:** Viktorija Serova, Mara Klibus, Zbignevs Marcinkevics, Uldis Rubins, Andris Grabovskis, Olegs Sabelnikovs

**Affiliations:** 1 Department of Anaesthesiology, Intensive Care and Clinical Simulations, Riga Stradins University, LV-1007 Riga, Latvia; 2 Faculty of Medicine and Life Sciences, University of Latvia, LV-1586 Riga, Latvia; 3 Faculty of Science and Technology, University of Latvia, LV-1586 Riga, Latvia

**Keywords:** septic shock, hemodynamic monitoring, microcirculation, capillary refill time, optical technologies, perfusion index

## Abstract

*Background and Objectives:* Septic shock is marked by profound circulatory and cellular dysfunction, with mortality rates of 25–40% despite guideline-based resuscitation. Normalization of macrohemodynamic variables often fails to restore tissue perfusion, a concept known as hemodynamic incoherence. Persistent microcirculatory dysfunction is associated with organ failure and poor outcomes, underscoring the limitations of systemic monitoring alone. This focused narrative review synthesizes current evidence on microcirculatory monitoring in septic shock, with emphasis on bedside and emerging optical technologies, and evaluates their role as adjuncts to traditional hemodynamic assessment for perfusion-targeted resuscitation. *Materials and Methods:* A concept-driven search of PubMed/MEDLINE (January 2015 to January 2026) was performed, incorporating MeSH and free-text terms for septic shock, microcirculation, hemodynamic coherence, and monitoring modalities. Foundational pre-2015 studies were included for context. Articles were screened using predefined inclusion/exclusion criteria to minimize bias, with thematic qualitative synthesis. A PRISMA-inspired flow diagram was used to summarize the study selection process. *Results*: Microcirculatory alterations in septic shock include reduced functional capillary density, perfusion heterogeneity, and impaired oxygen extraction, persisting despite macrohemodynamic correction. Bedside markers, such as capillary refill time (CRT) and mottling, track microvascular recovery more closely than lactate. When used to guide resuscitation, CRT-based strategies show a non-significant mortality trend in randomized evaluation, with later studies reporting benefit in composite clinical outcomes. Optical technologies offer non-invasive insights: photoplethysmography (PPG) and perfusion index (PI) show prognostic value and early detection of incoherence; automated CRT (aCRT) enhances reproducibility; advanced modalities, such as laser speckle contrast imaging (LSCI), near-infrared spectroscopy (NIRS), and sublingual videomicroscopy, provide detailed physiological data but face standardization challenges. Recent interventional evidence, including peripheral perfusion-targeted RCTs, supports improved outcomes, though large-scale trials remain limited. *Conclusions*: Microcirculatory monitoring provides complementary, physiologically relevant information to macrohemodynamic assessment in septic shock. Emerging bedside tools, such as PI and aCRT, are poised for routine use, while multimodal integration may enable personalized management. Future research should prioritize standardization, AI-driven analysis, and randomized trials to confirm outcome benefits.

## 1. Introduction

Septic shock remains one of the most severe and resource-intensive conditions encountered in critical care, with mortality consistently reported between 30 and 40% despite early recognition, antibiotics, fluid resuscitation, and vasopressor support [1]. Updated epidemiologic analyses underscore that sepsis-related mortality remains substantial despite advances in critical care and the urgent need for improved monitoring strategies. The Global Burden of Disease (GBD 2021) study estimated ~166 million sepsis episodes and approximately 13 million sepsis-associated deaths worldwide in 2021, corresponding to roghly one-fifth of all deaths globally, with a pronounced increase during 2020–2021 related to the COVID-19 pandemic [2]. A key driver of poor outcomes is hemodynamic incoherence, where macro-hemodynamic variables (e.g., mean arterial pressure [MAP], cardiac output [CO]) normalize, while microcirculatory perfusion remains severely impaired [3]. In a 2011 review, De Backer et al. highlighted that microcirculatory disturbances are thought to contribute substantially to the pathogenesis of sepsis-associated organ dysfunction [4].

Traditional perfusion assessment methods, such as capillary refill time (CRT), mottling score, lactate levels, passive leg raising (PLR), and echocardiographic indices, provide valuable insights but remain indirect measures of cellular oxygen delivery. CRT is simple yet subjective and sensitive to environmental factors [5], while lactate levels indicate global metabolic stress rather than real-time perfusion [6]. Echocardiography provides detailed macro-hemodynamic information but cannot visualize capillary blood flow and requires expertise.

The clinical importance of microcirculatory assessment is now recognized in recent guidelines. The November 2025 European Society of Intensive Care Medicine (ESICM) guidelines on Circulatory Shock and Hemodynamic Monitoring issued 50 recommendations, emphasizing serial tissue perfusion assessment and suggesting microcirculation evaluation as an adjunct when feasible [7]. This official endorsement underscores the growing consensus that tissues—not just systemic hemodynamic values—must guide shock resuscitation.

In response, optical technologies, such as photoplethysmography (PPG), remote PPG (rPPG), automated CRT (aCRT), laser speckle contrast imaging (LSCI), near-infrared spectroscopy (NIRS), and handheld videomicroscopy, have emerged as powerful tools capable of directly or indirectly assessing microvascular flow and perfusion heterogeneity. These modalities can augment traditional systemic monitoring and may enable more precise, physiologically targeted shock management.

### Aim of Focused Review

This focused review synthesizes current evidence on microcirculatory monitoring in septic shock and evaluates contemporary bedside methods, with particular emphasis on emerging optical technologies, including PPG, rPPG, aCRT, NIRS, and LSCI. The review explores how these tools may complement traditional hemodynamic monitoring, inform perfusion-targeted resuscitation, and support individualized clinical decision-making, while also addressing current gaps in standardization and outcome-driven research.

## 2. Materials and Methods

### 2.1. Review Design and Rationale

This manuscript was conducted as a focused narrative review using a structured, concept-driven approach, adapted from PRISMA 2020 guidelines to enhance transparency and reproducibility in non-systematic syntheses. Narrative methodology was intentionally chosen because the field of microcirculatory monitoring in septic shock is highly heterogeneous, encompassing diverse technologies, physiological concepts, and outcome measures that are not yet suitable for quantitative meta-analysis. Emerging optical modalities often lack standardized endpoints or large randomized trials, limiting the feasibility and interpretability of a systematic review or meta-analysis. The objective, therefore, was to integrate pathophysiological principles, clinical evidence, and technological advances into a coherent framework relevant to bedside decision-making.

### 2.2. Literature Search Strategy

A targeted literature search was conducted across PubMed/MEDLINE, covering publications from January 2015 to January 2026. Foundational studies pre-2015, identified via reference lists of included articles and guidelines, were incorporated for essential pathophysiological context (e.g., De Backer 2011 [4] on microcirculatory mechanisms). The strategy used Medical Subject Headings (MeSH) and free-text terms related to septic shock, tissue perfusion, and microcirculatory monitoring. Key search terms included combinations of the following terms:

*septic shock, sepsis, microcirculation, hemodynamic coherence, tissue perfusion, capillary refill time, automated capillary refill time, photoplethysmography, perfusion index, remote photoplethysmography, laser speckle contrast imaging, near-infrared spectroscopy, sublingual microcirculation, incident dark field imaging, lactate, mottling, passive leg raising, and echocardiography.*

Boolean operators (AND/OR) were applied as appropriate. Reference lists of key reviews, landmark trials, and international guidelines (e.g., 2025 ESICM [7]) were manually screened to identify additional relevant studies.

### 2.3. Study Selection and Eligibility Criteria

**Figure 1:**
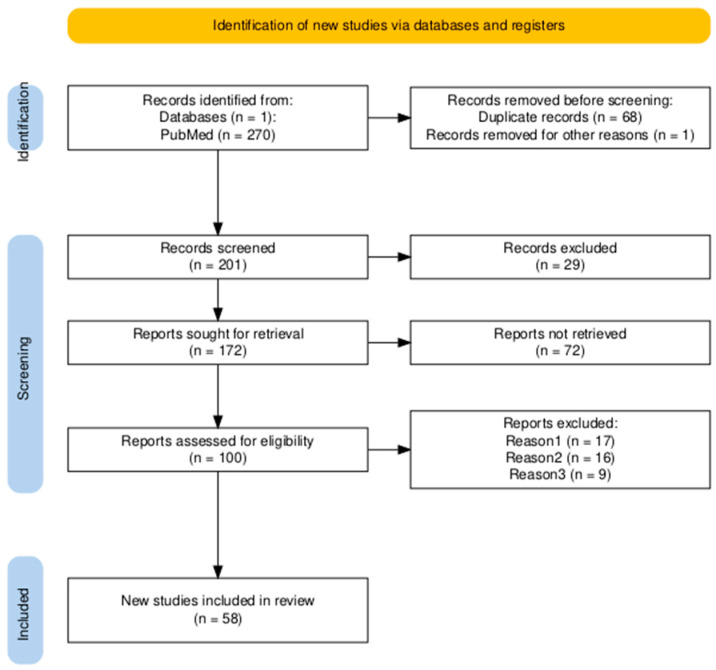
The PRISMA 2020-inspired flow diagram for article selection [8].

Although not a formal systematic review, a PRISMA-inspired process was used to minimize selection bias *(*Figure 1). Nevertheless, the authors used a structured, transparent approach to identifying and selecting the literature. To minimize selection bias and improve completeness, the authors predefined inclusion/exclusion criteria. Two authors independently screened titles/abstracts, then full texts, resolving discrepancies through discussion.

Articles were included if they met one or more of the following criteria:

- Addressed microcirculatory dysfunction in adult sepsis or septic shock;
- Evaluated bedside or optical technologies for perfusion or microcirculatory assessment;
- Provided clinical, physiological, or translational insights relevant to hemodynamic coherence;
- Reported findings from major randomized trials, observational studies, or international guidelines related to perfusion monitoring;
- High-quality conceptual and narrative reviews by recognized experts were included when they contributed to theoretical integration or contextual interpretation of the evidence.

Exclusion Criteria

- Studies were excluded if they
- Were purely experimental or animal-based without clear clinical relevance;
- Focused solely on engineering or signal-processing development without clinical validation;
- Included exclusively pediatric populations;
- Represented duplicate publications or secondary analyses of the same patient cohort;
- Were unrelated to tissue perfusion, microcirculation, or shock physiology.

Approximately 270 unique records were identified; after screening, 58 were included. The final reference set includes 4 randomized controlled trials, over 20 observational clinical studies, more than 15 reviews/meta-analyses, and multiple international consensus guidelines, ensuring representation of experimental, physiological, and expert-consensus evidence across the spectrum of septic shock and microcirculatory monitoring.

### 2.4. Data Synthesis

Data were qualitatively synthesized and organized into thematic domains, encompassing microcirculatory pathophysiology, conventional clinical perfusion markers, optical and imaging-based monitoring techniques, clinical outcome associations, and implementation challenges. No formal risk-of-bias assessment was performed due to the narrative scope, but study quality was considered (e.g., prioritizing RCTs like ANDROMEDA-SHOCK-2 over observational studies). More recent evidence (post-2025) was incorporated to address gaps in interventional trials. Emphasis was placed on clinical applicability, physiological plausibility, and consistency of findings across studies.

## 3. Rationale for Integrating Microcirculatory Monitoring and Basics of Microcirculation in Septic Shock

Recent studies highlighted the emerging importance of integrating microcirculatory assessment into the hemodynamic management of septic shock. De Backer et al. emphasized that novel microvascular monitoring tools, such as automated imaging systems, simplified bedside devices, and emerging optical technologies, enable routine evaluation of microvascular flow and tissue perfusion at the bedside, potentially overcoming many of the technical limitations that previously restricted their clinical use [9]. On the other hand, Bakker and Ince underscored the concept of hemodynamic coherence, noting that restoration of systemic parameters (e.g., MAP) does not necessarily translate into microcirculatory recovery, and that persistent discordance between macro- and microcirculation is associated with worse outcomes (e.g., organ failure rates up to 50%) [10]. A scoping review of vasoactives in septic shock found microcirculatory improvements aligned with cardiac index in only 39% of studies, emphasizing variability with agents such as norepinephrine [11]. Similarly, fluid administration’s effects on sublingual microcirculation vary, with albumin potentially superior to crystalloids in select cases, though evidence remains mixed due to methodological artifacts [12]. These perspectives suggest that future resuscitation strategies should aim not only to correct systemic hemodynamic but also to ensure microvascular reperfusion, combining traditional monitoring with targeted assessment of tissue-level perfusion to better individualize therapy in septic shock, potentially enhanced by AI for real-time analysis. Hemodynamic variables of key quantitative microcirculatory and peripheral perfusion parameters in health and septic shock are reported in Table 1.

These parameters help quantify dysfunction severity and evaluate interventions, and meta-analyses confirm strong prognostic associations between impaired microcirculatory parameters and mortality. The microcirculation is composed of arterioles, capillaries, and venules that regulate oxygen delivery, nutrient transport, and metabolic exchange. Under normal conditions, microvascular units dynamically adjust blood flow according to tissue metabolic needs, ensuring relatively uniform perfusion. In septic shock, profound pathophysiologic changes occur. Systemic inflammation causes endothelial injury, increased permeability, vasoplegia, and dysregulated nitric oxide signaling [31]. Glycocalyx degradation contributes to capillary leak and loss of shear-dependent vasoregulation [32]. Coagulation abnormalities promote microthrombi formation, contributing to capillary obstruction and heterogeneous perfusion [33].

These mechanisms reduce functional capillary density and increase perfusion heterogeneity, core quantitative parameters summarized in Table 1, and impair tissue oxygen extraction [15,16]. As a result, even when systemic hemodynamic variables appear normal, cellular hypoxia may persist. Sublingual videomicroscopy consistently demonstrates that persistent microcirculatory derangements correlate with organ dysfunction and increased mortality, with impaired microvascular flow indices repeatedly observed in non-survivors [4,17,34]. Recent cohort data reinforce sublingual monitoring’s role in assessing resuscitation efficacy, with improvements predicting better outcomes [35]. Integration of quantitative microcirculatory metrics with advanced analytical tools may support future personalized resuscitation strategies and help bridge the bench-to-bedside gap.

## 4. Routinely Used Markers

Conventional markers remain essential for perfusion assessment in septic shock, providing indirect insights into tissue perfusion. However, their limitations in capturing real-time microvascular dynamics support integration with advanced monitoring tools. Observational studies and randomized trials indicate that conventional perfusion markers correlate with outcomes, yet they do not consistently reflect the timing or heterogeneity of microcirculatory recovery.

### 4.1. Lactate Levels

Lactate remains a central biomarker in septic shock resuscitation, yet it represents a complex global signal rather than a direct indicator of microcirculatory perfusion. Elevated lactate may arise from tissue hypoxia but also from β-adrenergic-driven glycolysis, impaired hepatic clearance, and mitochondrial dysfunction, limiting its specificity for assessing regional oxygen delivery [6]. Contemporary microcirculation research demonstrates that substantial alterations in functional capillary density and perfusion heterogeneity may persist despite lactate normalization, indicating a measurable dissociation between systemic lactate kinetics and microvascular recovery [3,10,15,16]. These physiological considerations have shaped the evolution of clinical trial design, in which lactate has been evaluated both as an active resuscitation target and as a limited biomarker of tissue perfusion. In ANDROMEDA-SHOCK (2019), lactate was used as a direct treatment target and compared with CRT-guided resuscitation, demonstrating comparable outcomes with lower fluid and vasopressor exposure and a non-significant trend toward reduced 28-day mortality (HR 0.75; 95% CI 0.55–1.02). In contrast, ANDROMEDA-SHOCK-2 (2025) trial was informed by recognition of lactate’s limitations as a sole perfusion marker and evaluated a CRT-targeted personalized resuscitation strategy against usual care, demonstrating benefit in a hierarchical composite outcome driven primarily by reduced duration of vital organ support [5,36]. Additional observational data indicate weak to moderate correlations between systemic lactate and peripheral perfusion indices, such as perfusion index (PI), mottling, and CRT, and demonstrate that microcirculatory abnormalities may persist despite apparently adequate lactate levels [22,23,37,38,39]. Collectively, current evidence supports using lactate as a marker of systemic metabolic stress while integrating it with microcirculatory assessments to obtain a more accurate evaluation of tissue-level perfusion in septic shock.

### 4.2. Capillary Refill Time (CRT)

Capillary refill time (CRT) has re-emerged as a clinically accessible bedside marker of peripheral perfusion in septic shock, reflecting the balance between local microvascular flow, vasomotor tone, and intravascular volume status [40]. Compared with biochemical markers, such as lactate, a delayed and non-specific global metabolic signal, CRT offers an immediate assessment of peripheral perfusion, responding dynamically to changes in microcirculatory blood flow and perfusion pressure. From a guideline perspective, CRT is best viewed as a complementary perfusion marker rather than a standalone target.

In this context, clinical trial evidence positions CRT as a practical bedside trigger within a multimodal resuscitation strategy rather than a standalone therapeutic endpoint. The ANDROMEDA-SHOCK (2019) program incorporated CRT normalization within routine bedside assessment to guide early resuscitation decisions in septic shock, thereby establishing CRT as a repeatable clinical target and demonstrating the feasibility of peripheral perfusion-guided decision-making at the bedside. Building on this concept, ANDROMEDA-SHOCK-2 (2025) embedded CRT within a personalized resuscitation framework aligned with usual care, reflecting an evolution from single-marker targeting toward integrated perfusion assessment. Collectively, these trials support the implementation value of CRT-guided approaches while reinforcing the importance of integrating CRT with systemic, biochemical, and organ-level parameters rather than relying on any single perfusion marker in isolation [5,36,37].

Observational studies further show that prolonged CRT is associated with hyperlactatemia, organ dysfunction, and increased mortality risk, reinforcing its prognostic relevance in circulatory shock [41]. Advances in quantitative and automated CRT methods (aCRT, Q-CRT) have improved measurement reproducibility and reduced operator dependency, with superior diagnostic performance for identifying higher-risk patients, particularly in early sepsis and suspected infection [42]. Overall, contemporary evidence supports CRT as a valid, rapid, non-invasive indicator of peripheral perfusion status that complements systemic biomarkers in perfusion-guided resuscitation strategies in septic shock.

### 4.3. Mottling Skin

Skin mottling—a patchy, uneven discoloration of the knee and thigh regions—reflects peripheral hypoperfusion and microcirculatory impairment in septic shock. Although originally described decades ago, interest in mottling as a clinical perfusion marker has grown as clinicians seek bedside indicators of microvascular dysfunction beyond systemic parameters. Semiquantitative mottling scores, typically ranging from 0 (no mottling) to 5 (extensive involvement), have demonstrated prognostic value: in a large observational study, mottling score independently predicted 14-day mortality across vasopressor doses and remained significant under Sepsis-3 definitions; moreover, a decrease in mottling score during resuscitation was associated with better outcomes (OR 2.26 [95% CI 1.72–2.97] for mortality in the multivariable model) [43]. In addition, observational data indicate that higher mottling scores are associated with reduced skin microcirculatory oxygen saturation, indicating that more severe mottling reflects greater microvascular derangement [38]. Systematic evaluations of bedside peripheral perfusion markers further confirm that mottling, alongside CRT and perfusion index (PI), serves as a convenient, non-invasive indicator of peripheral perfusion abnormalities and mortality risk in septic populations, although sensitivity and specificity are moderate and should be interpreted alongside other perfusion data [39]. Thus, the mottling score remains a clinically useful marker of microcirculatory impairment in septic shock and an accessible tool for perfusion assessment at the bedside.

### 4.4. Passive Leg Rise

Passive leg raising (PLR) is a reversible bedside maneuver that transiently increases venous return and mimics an “internal fluid challenge,” allowing clinicians to assess fluid responsiveness without administering intravenous fluids. In critically ill patients with septic shock, a positive PLR test (e.g., >10% stroke volume increase) reliably identifies those who are likely to improve hemodynamically with fluid administration, with meta-analyses reporting high sensitivity and specificity for predicting fluid responsiveness, thereby helping tailor early resuscitation. Meta-analyses confirm that PLR-induced changes in cardiac output predict fluid responsiveness with high sensitivity and specificity, providing a dynamic assessment that integrates both macro- and microhemodynamic determinants of perfusion [44]. A contemporary review further emphasizes PLR as a cornerstone dynamic test for predicting fluid responsiveness in acute circulatory failure, while highlighting key methodological prerequisites (e.g., real-time cardiac output tracking) that may explain variability across clinical settings [45]. Although direct studies focused exclusively on microcirculatory parameters in septic shock patients using PLR are limited in the recent literature, available data largely rely on microvascular surrogates rather than sublingual videomicroscopy, including near-infrared spectroscopy (NIRS)-derived indices and plethysmography-based perfusion metrics. Foundational evidence demonstrates that maneuvers increasing preload, such as PLR and subsequent fluid challenge, are associated with improvements in sublingual microvascular perfusion indices (e.g., functional capillary density, proportion of perfused vessels, microvascular flow index) in patients with sepsis and septic shock, suggesting that PLR-triggered increases in central blood volume may translate into enhanced peripheral microcirculatory perfusion when preload responsiveness is present [46]. Taken together, PLR serves as a useful physiologic test in septic shock to assess fluid responsiveness and, when integrated with microcirculatory evaluation tools (e.g., PI, sublingual imaging), enhances understanding of the interplay between macro- and microcirculatory perfusion during early resuscitation.

### 4.5. Transthoracic Echocardiography (TTE)

Echocardiography is a cornerstone of hemodynamic evaluation in septic shock, providing real-time assessment of cardiac output, preload dependency, and ventricular function. However, echocardiographic normalization of macrohemodynamic parameters does not necessarily reflect adequate microcirculatory perfusion, with persistent macro-microcirculatory incoherence in 30–50% of cases. Some studies emphasize that markers of peripheral perfusion, such as capillary refill time (CRT), mottling score, and perfusion index (PI), may remain abnormal even when echocardiographic indices suggest sufficient cardiac output or fluid responsiveness [47]. Integration of echocardiography with bedside perfusion markers, therefore, offers complementary information: TTE identifies the mechanism of shock (e.g., distributive vs. cardiogenic), while peripheral microcirculation assessments reveal the effectiveness of tissue-level perfusion and reperfusion strategies. Point-of-care ultrasound (POCUS) extensions may improve fluid responsiveness assessment in sepsis, but macrocirculatory responsiveness does not consistently translate to microcirculatory recovery (e.g., inferior vena cava variability predicts perfusion gains). Recent guideline updates also highlight that echocardiography should be used as part of a multimodal hemodynamic assessment, in conjunction with peripheral perfusion monitoring to guide individualized resuscitation rather than as a standalone determinant of perfusion adequacy [7].

## 5. Optical Devices for Microcirculation Evaluation

Optical technologies have advanced rapidly, offering non-invasive or minimally invasive methods to assess microvascular flow, heterogeneity, and oxygenation in septic shock. Observational studies consistently demonstrate prognostic associations between impaired optical microcirculatory indices and adverse outcomes, although interventional validation remains limited. Recent innovations, such as optical coherence tomography angiography (OCTA) and hyperspectral imaging, expand the toolkit, with proof-of-concept studies showing feasibility for real-time bedside use. These approaches complement conventional perfusion markers by providing earlier and more direct insight into microvascular dysfunction and hemodynamic incoherence. Key features are summarized in Table 2.

### 5.1. Photoplethysmography (PPG)

PPG measures pulsatile changes in tissue blood volume using reflected light. The perfusion index (PI) represents the ratio of pulsatile to non-pulsatile components of the signal. Low PI reflects vasoconstriction or hypoperfusion. Several studies show that PI correlates with vasopressor requirement (reported AUC up to 0.96),markers of illness severity, organ dysfunction, and mortality in critically ill cohorts [23,24,25]. PI also improves earlier than lactate in surgical and perioperative critically ill populations, making it a dynamic marker of perfusion recovery [26]. Recent machine-learning models applied to raw PPG waveforms significantly enhanced early sepsis detection accuracy, achieving AUC values >0.80 and outperforming conventional vital-sign-based approaches [27].

Perfusion index (PI) represents beat-to-beat changes in local arterial pulsatility and should be viewed as a peripheral perfusion signal rather than a direct measure of organ microvascular flow [28,29]. Physiological and clinical studies demonstrate that, because PI is strongly influenced by vasomotor tone and systemic conditions (e.g., temperature and sympathetic activation), it should be interpreted as a composite marker of peripheral perfusion rather than a specific measure of microvascular flow [29]. In septic shock, PI can still capture clinically meaningful aspects of microcirculatory behaviour: following fluid resuscitation, PI-based reactive hyperaemia testing shows blunted hyperaemic responses, consistent with impaired microvascular reactivity, although correlations with global markers are modest [22]. Conversely, norepinephrine titration studies show that increasing mean arterial pressure often raises PI, but with substantial inter-individual variation, indicating that PI during vasopressor therapy primarily reflects the interaction of perfusion pressure and vasomotor tone rather than pure microvascular perfusion [30]. Overall, PI and PPG pulse waveforms are reasonable, non-invasive tools for tracking dynamic changes in peripheral perfusion and may serve as useful surrogates of microcirculatory status over time. However, PI should be interpreted alongside other perfusion markers, such as lactate, capillary refill time, and organ function, because it does not directly measure microvascular blood flow.

### 5.2. Remote Photoplethysmography (rPPG)

rPPG extracts subtle color variations from video recordings of skin, allowing contactless measurement of blood-flow-related pulsatility. Foundational studies established the feasibility of extracting pulsatile physiological signals from video-based skin imaging [48]. rPPG is particularly useful in patients where sensor placement is difficult (burns, edema) or infection-control scenarios, as it enables physiological monitoring without direct skin contact [49]. Supporting early clinical feasibility, a recent ICU case series in septic shock showed that rPPG waveform features varied in parallel with vasopressor titration and arterial pressure, suggesting indirect sensitivity to peripheral microcirculatory dynamics [50]. Limitations include environmental sensitivity, and emerging multimodal devices improve applicability.

### 5.3. Automated Capillary Refill Time (aCRT)

Automated capillary refill time measurements (often termed quantitative or automated CRT, Q-CRT/aCRT). Use controlled mechanical compression and optical detection (pulse oximetry or camera-based systems) to standardize the stimulus and objectively quantify reperfusion time, reducing operator dependence compared with manual. In critically ill patients, Q-CRT measured with a pulse oximetry-based device shows moderate-to-strong correlation with blood lactate (r = 0.7) and good discrimination for hyperlactatemia (AUC 0.85), supporting its role as a surrogate of systemic hypoperfusion [51]. In emergency department sepsis screening, Q-CRT demonstrated comparable predictive performance to lactate and qSOFA and improved sensitivity when combined with qSOFA (combined AUC approaching 0.9), linking automated CRT indices to sepsis severity and shock risk [42]. Recent point-of-care systems that integrate standardized fingertip compression with infrared sensing further improve precision and reproducibility relative to visual CRT, offering a path toward less variable bedside perfusion assessment [52].

### 5.4. Laser Speckle Contrast Imaging

Laser Speckle Contrast Imaging (LSCI) is a contactless optical technique that illuminates tissue with coherent laser light and analyzes the temporal blurring of the speckle pattern fluctuations to generate real-time, two-dimensional maps of superficial skin blood flow. Clinically, this enables high-resolution assessment of cutaneous microcirculation across a relatively large field of view, supporting evaluation of spatial perfusion heterogeneity rather than single-point measurement. In septic shock, LSCI has been applied to fingertip microcirculation. In a prospective study of 44 patients with sepsis and septic shock, Ruan et al. used LSCI-derived perfusion index to quantify skin blood flow and showed that septic shock patients had significantly lower perfusion indices than septic patients, that these indices discriminated sepsis vs shock (AUC 0.8), and that lower values correlated with higher APACHEII and SOFA scores and moderately with lactate and worse outcome, suggesting that LSCI can capture the severity of microcirculatory impairment and provide prognostic information in this population [53]. Beyond sepsis, a 2024 iScience study in undifferentiated shock demonstrated that non-contact LSCI of peripheral regions of interest detected dynamic changes in skin blood flow and achieved diagnostic performance around AUC ≈ 0.8, while enabling rapid bedside acquisition compared with conventional assessment approaches [54].

Methodological reviews emphasize that LSCI offers fast, full-field, repeatable imaging of superficial microvascular perfusion in diverse clinical settings, including critical illness, but they also underline important limitations: Speckle-derived perfusion units are semi-quantitative and mainly suited for relative rather than absolute blood-flow comparisons; the technique is restricted to superficial tissues (skin of exposed organs) and may not directly reflect deeper organ perfusion; measurements are sensitive to motion, ambient light and acquisition settings (exposure time, distance, angle). In addition, limited standardized protocols and calibration restrict comparability between devices and centers [55]. Thus, in septic shock, LSCI should be viewed as a promising investigational adjunct for peripheral microcirculatory assessment and tissue perfusion [56], particularly for risk stratification and monitoring response to therapy. Current use remains largely investigational, requiring integration with conventional macrohemodynamics and biochemical markers and further validation before routine incorporation into hemodynamic monitoring algorithms.

### 5.5. Near-Infrared Spectroscopy (NIRS)

Near-Infrared Spectroscopy (NIRS) is a non-invasive optical method that emits near-infrared light (700–900 nm) into tissue and measures the attenuation due to absorption by oxygenated and deoxygenated hemoglobin, thereby estimating local tissue oxygen saturation (StO_2_). In critically ill or septic patients, NIRS enables continuous bedside monitoring of regional tissue oxygenation. Most commonly in peripheral muscles (thenar, forearm, leg) or skin, providing dynamic information on tissue perfusion and oxygen delivery at the microvascular level. Clinical studies have shown that impaired NIRS-derived STO_2_ or altered microvascular reactivity on NIRS-based vascular occlusion tests (VOT) correlate with tissue hypoperfusion and are associated with worse clinical outcomes in circulatory shock, supporting their role as markers of impaired perfusion [18]. Because NIRS provides a global measure over a tissue bed rather than sampling a single capillary, it captures averaged oxygenation and perfusion trends, making it useful for monitoring response to therapy (fluids, vasopressors) or detecting early deterioration [19]. However, NIRS also has important limitations: its measurement depth is limited to superficial tissues (only a few millimeters), it reflects primarily superficial microcirculation (skin or muscle) and may not represent perfusion of internal organs; StO_2_ depends not only on flow, but also on local metabolic consumption and hemoglobin concentration; signal quality is affected by skin pigmentation, probe placement, oedema, and ambient light; and the lack of standardization (probe site, baseline values, thresholds) hampers generalizability between patients and centers [20]. Thus, while NIRS is a promising adjunct for non-invasive, bedside microcirculatory monitoring and perfusion assessment in septic shock, it should be interpreted cautiously and preferably in conjunction with other perfusion and hemodynamic indicators [21].

### 5.6. Sublingual Videomicroscopy

Handheld sublingual videomicroscopy using incident dark field (IDF) or sidestream dark-field (SDF) imaging provides direct, in vivo visualization of the microcirculation, enabling quantification of functional capillary density, flow heterogeneity, and microvascular flow index in critically ill patients. Because the sublingual mucosa is easily accessible and shares embryologic and hemodynamic characteristics with the splanchnic circulation, it is considered a clinically relevant window into systemic microcirculatory dysfunction. In septic shock, sublingual videomicroscopy has demonstrated that decreases in capillary density, increases in perfusion heterogeneity, and the presence of stagnant or intermittently flowing capillaries correlate strongly with organ dysfunction and mortality, even when macrocirculatory variables, such as MAP, are within target ranges [17]. Technological advances, particularly newer-generation IDF devices, have improved image quality and contrast, enabling more reliable automated or semi-automated quantification of microvascular parameters [57]. A 2024 meta-analysis confirmed that impaired sublingual microcirculation is independently associated with higher mortality and illness severity in sepsis, underscoring its prognostic value [34]. Despite its strengths, practical limitations continue to restrict routine clinical implementation. Image acquisition requires training and is vulnerable to motion artifacts and pressure-related distortion; analysis may be time-consuming and remains incompletely standardized across devices; and microcirculatory status may vary among different vascular beds, limiting extrapolation from the sublingual region to internal organs. Despite these constraints, sublingual videomicroscopy remains the most mature and widely studied method for direct bedside assessment of microvascular function in septic shock and continues to play a central role in research focused on hemodynamic coherence and perfusion-targeted resuscitation.

### 5.7. Emerging Optical Modalities

Emerging optical technologies are expanding the scope of bedside perfusion assessment beyond conventional sublingual videomicroscopy and peripheral perfusion surrogates. Optical coherence tomography angiography (OCTA) enables high-resolution, depth-resolved perfusion mapping and has been explored for retinal microvascular assessment in sepsis, with feasibility work extending to registered clinical studies. (e.g., clinicaltrials.gov NCT04593212). Hyperspectral imaging (HSI) provides non-contact, bedside quantification of superficial tissue oxygenation and hemoglobin-related surrogates, and preliminary clinical data in sepsis suggest that HSI can identify characteristic microcirculatory/perfusion patterns, supporting further evaluation of this modality for monitoring tissue perfusion during critical illness [58].

## 6. Discussion

The focused narrative review synthesizes evidence from 58 studies, highlighting that microcirculatory dysfunction is a central pathophysiological feature of septic shock and frequently persists despite apparent normalization of macrohemodynamic variables. This dissociation, described as hemodynamic incoherence, explains why conventional systemic parameters, such as mean arterial pressure or cardiac output, may fail to reflect true tissue perfusion and oxygen delivery [2,8]. Growing clinical and experimental evidence indicates that persistent microvascular alterations—characterized by reduced functional capillary density (e.g., from 16.6 ± 1.6 mm/mm^2^ in healthy to 13.2 ± 4.4 mm/mm^2^ in septic shock, representing a ~20–30% relative reduction) lower in non-survivors, heterogeneous flow distribution (PHI 0.3–0.5), and impaired oxygen extraction—are closely associated with organ dysfunction and adverse outcomes in septic shock [4,14].

Traditional bedside markers of perfusion, including serum lactate, capillary refill time (CRT), mottling score, passive leg raising, and transthoracic echocardiography, remain fundamental components of shock assessment [5,15]. However, each of these parameters provides only indirect or partial insight into microcirculatory function. Lactate reflects global metabolic stress rather than real-time regional perfusion, while echocardiography primarily assesses macrohemodynamic determinants of flow [38]. In contrast, peripheral perfusion markers, such as CRT and mottling, demonstrate closer temporal coherence with changes in peripheral perfusion and have been associated with clinical outcomes and response to resuscitation. This concept is supported by clinical trial evidence from the ANDROMEDA-SHOCK (2019) and ANDROMEDA-SHOCK-2 (2025), which evaluated CRT-guided strategies using distinct but complementary designs. In ANDROMEDA-SHOCK (2019), CRT-guided resuscitation was directly compared with lactate-guided therapy and resulted in similar overall clinical outcomes, with lower fluid and vasopressor exposure, and a non-significant trend toward reduced 28-day mortality (HR 0.75; 95% CI 0.55–1.02). Building on recognition of lactate’s limitations as a sole perfusion target, ANDROMEDA-SHOCK-2 (2025) evaluated a CRT-targeted personalized resuscitation strategy against usual care and demonstrated superiority in a hierarchical composite outcome (win ratio 1.16; 95% CI 1.02–1.33), driven primarily by a shorter duration of vital organ support, without a significant mortality difference (HR 0.99; 95% CI 0.81–1.21) [5,36]. Collectively, these findings support the concept that bedside assessment of tissue perfusion can complement systemic hemodynamic monitoring and may help avoid overtreatment driven solely by delayed biochemical markers.

In recent years, optical technologies have emerged as promising tools for non-invasive or minimally invasive evaluation of microcirculatory status [56]. Among these, photoplethysmography-derived perfusion index (PI) and automated capillary refill time (aCRT) are closest to routine clinical applicability due to their simplicity, low cost, and ease of integration into existing monitoring systems. These modalities provide continuous or standardized assessments of peripheral perfusion and may facilitate dynamic monitoring of resuscitation effects [22,46]. Nevertheless, they remain indirect surrogates of microvascular flow and are influenced by systemic hemodynamics, vasomotor tone, temperature, and local tissue conditions [43].

More advanced techniques, including laser speckle contrast imaging (LSCI), near-infrared spectroscopy (NIRS), and sublingual videomicroscopy, offer richer physiological information by assessing microvascular flow or tissue oxygenation more directly [26,28]. Sublingual videomicroscopy remains the most established method for in vivo visualization of microcirculation and has demonstrated strong associations with organ failure and mortality [14,49]. However, its clinical implementation is limited by technical complexity, need for expertise, and lack of standardized analysis protocols [14,50]. Similarly, LSCI and NIRS provide valuable insights into peripheral microcirculatory perfusion but are currently constrained by variability in measurement conditions, limited penetration depth, and insufficient outcome-driven validation [26,30]. Emerging options, such as OCTA/hyperspectral, show promise for high-resolution mapping, with early observational studies suggesting potential for detecting microcirculatory alterations [58]. A 2026 vasoactives review notes agent-specific variability (e.g., norepinephrine alignment in 39%), underscoring personalized needs [11].

Importantly, recent international guidelines, including the 2025 ESICM recommendations, acknowledge the clinical relevance of tissue perfusion assessment and recognize microcirculation monitoring as a potential adjunct to hemodynamic evaluation when feasible [7]. At present, however, the evidence base remains insufficient to support routine microcirculation-guided resuscitation strategies [7,8]. High-quality randomized trials assessing whether interventions guided by microcirculatory parameters improve patient-centered outcomes are still lacking [7].

Overall, the available data suggest that microcirculatory monitoring should be viewed as complementary to, rather than a replacement for, conventional hemodynamic assessment. A multimodal approach that integrates macrohemodynamics, peripheral perfusion markers, and selected microcirculatory tools may offer the most pragmatic pathway toward more individualized management of septic shock [4].

### 6.1. Challenges in Implementation

Despite strengths, barriers persist: Standardization is lacking (e.g., videomicroscopy artifacts in 20–30% of acquisitions), limiting comparability; the majority ofavailable evidence remains observational, with relatively few RCTs (e.g., only PI-targeted trials show outcome benefits). Logistical issues (training, cost) restrict routine use, and superficial focus may not reflect visceral perfusion. Bias risks in narrative synthesis (e.g., publication bias toward positive associations) were mitigated via dual screening, but formal meta-analysis awaits field maturation.

### 6.2. Clinical Implications and Multimodal Integration

Microcirculatory monitoring complements macrohemodynamics, enabling tissue-oriented resuscitation. For instance, CRT/PI-guided strategies reduce fluids by 20–30% while improving coherence. Guidelines endorse adjunctive use when feasible, but evidence gaps hinder routine adoption. A multimodal approach—combining conventional (CRT/lactate) with optical (PI/NIRS/videomicroscopy)—offers pragmatism, as in the PRISM trial protocol, which personalizes via real-time integration. AI could automate analysis (e.g., PPG waveform AUC > 0.80), fostering precision medicine.

### 6.3. Future Direction

Research priorities include (1) standardization protocols for comparability; (2) large RCTs assessing micro-guided interventions on patient-centered outcomes (e.g., mortality, organ support days); (3) multimodal/AI platforms for coherence monitoring; and (4) validation of emerging tools (OCTA/hyperspectral) in diverse cohorts. Perspectives from 2026 reviews emphasize personalization, potentially reducing septic shock’s 30–40% mortality through bench-to-bedside translation. Until robust evidence emerges, microcirculatory tools should be adjunct to comprehensive assessment, not replace it.

## 7. Conclusions

Microcirculatory dysfunction is a hallmark of septic shock and plays a key role in the development of organ failure and adverse outcomes. Conventional systemic hemodynamic parameters alone are insufficient to fully characterize tissue perfusion, underscoring the clinical relevance of assessing the microcirculation. Bedside markers, such as capillary refill time and mottling, provide accessible information on peripheral perfusion, while emerging optical technologies offer additional insight into microvascular flow and tissue oxygenation.

Among currently available modalities, peripheral perfusion-oriented tools, such as photoplethysmography-derived indices and automated capillary refill time, appear closest to clinical implementation. Evidence from ANDROMEDA-SHOCK (2019) and ANDROMEDA-SHOCK-2 (2025) supports targeting peripheral perfusion within a multimodal resuscitation framework, with benefits reflected in composite outcomes and reduced organ support rather than consistent mortality effects, favoring integrated bedside perfusion assessment.

Advanced imaging techniques, such as sublingual videomicroscopy, laser speckle contrast imaging, and near-infrared spectroscopy, remain primarily research tools.

Innovations such as OCTA and hyperspectral imaging hold promise for high-resolution, AI-enhanced monitoring.

Although these modalities illuminate hemodynamic incoherence, their impact on outcomes remains unproven in large trials. Clinicians should view microcirculatory assessment as an adjunct to multimodal hemodynamic strategies, integrating tools such as PI/CRT with TTE/lactate for personalized resuscitation.

Future research should focus on standardization of measurement protocols, AI integration of multimodal real-time perfusion monitoring, and well-designed interventional trials to determine whether microcirculation-guided strategies can improve outcomes in septic shock. Until such evidence is available, microcirculatory monitoring should be considered an adjunct to comprehensive hemodynamic assessment rather than a standalone target for resuscitation.

## Figures and Tables

**Table 1:** Bedside microcirculatory and peripheral perfusion parameters in health and septic shock.

Parameter	Definition	Units	Normal Value (Healthy Adults) *	Typical Pattern in Septic Shock *	Prognostic Notes	Sources
Functional Capillary Density (FCD)	Total length of continuously perfused capillaries per tissue area	mm/mm^2^	~16–18	A decrease of ~20–30% from normal	Persistent reduction associated with organ failure and mortality	[3,13,14,15,16]
Proportion of Perfused Vessels (PPV)	Percentage of small vessels with continuous/sluggish flow	%	>95–99%	~50–80%	PPV < 80% linked to severe microvascular dysfunction and mortality	[3,13,14,15,16]
Microvascular Flow Index (MFI)	Semi-quantitative microvascular flow score (0–3 scale)	Score	≥2.9	~1.5–2.2	MFI < 2 associated with worse outcomes	[3,14,15,17]
Perfusion Heterogeneity Index (PHI)	Spatial variability of microvascular perfusion	Ratio	<0.2	0.3–0.5	Increased heterogeneity predicts mortality	[3,14,16]
Tissue Oxygen Saturation (StO_2_, NIRS)	Regional tissue oxygen saturation measured by near-infrared spectroscopy	%	~75–85% (site/device dependent)	Often < 70%; highly variable	Reflects impaired oxygen extraction and tissue hypoxia	[18,19,20,21]
Perfusion Index (PI, PPG)	Ratio of pulsatile to non-pulsatile photoplethysmographic signal amplitude	Dimensionless	Wide physiologic range; typically, >1 in well-perfused adults	Often < 1 with peripheral vasoconstriction	Low PI associated with vasopressor requirement and poor peripheral perfusion	[22,23,24,25,26,27,28,29,30]

* Values may vary depending on device, measurement site, and clinical context; ranges are approximate and derived from observational septic shock cohorts.

**Table 2:** Comparative overview of optical technologies.

Technology	Principle	Penetration Depth *	Clinical Readiness	Strengths	Limitations	Supporting Studies (N, Recent Examples)
PPG/PI	Pulsatile blood volume changes via reflected light	~1–2 mm	High (routine monitors)	Continuous, low-cost; early sepsis detection (AUC > 0.80)	Influenced by vasomotor tone; indirect	7 (e.g., machine-learning models, 2022–2025)
rPPG	Contactless color variations from video	Superficial (~1 mm)	Moderate (infection control)	Non-contact; correlates with hemodynamics (r = 0.7)	Sensitive to lighting/motion	4 (e.g., fluid response studies, 2016–2025)
aCRT/Q-CRT	Automated compression/optical reperfusion timing	Superficial	High (point-of-care devices)	Reproducible (AUC 0.85 for shock); better than manual	Limited to periphery	6 (e.g., ED sepsis screening, 2019–2025)
LSCI	Speckle pattern fluctuations for blood flow maps	~1 mm	Moderate (investigational)	Full-field, real-time; prognostic in shock (AUC 0.8)	Semi-quantitative; motion-sensitive	8 (e.g., sepsis discrimination, 2022–2025; microclots via LSI/LDI)
NIRS	Near-infrared absorption for tissue oxygenation (StO_2_)	1–3 cm	Moderate	Continuous regional monitoring: VOT reactivity predicts outcomes (OR 2.5)	Shallow depth; affected by edema/pigmentation	12 (e.g., impaired StO2 in shock, 2016–2024)
Sublingual Videomicroscopy (IDF/SDF)	Direct capillary visualization	Superficial	Low (research)	Detailed (e.g., MFI < 2.0, mortality OR 3.1); prognostic meta-analysis	Operator-dependent; artifacts	15 (e.g., 2024 meta; resuscitation efficacy cohorts, 2025)
Emerging (e.g., OCTA, Hyperspectral)	Coherence tomography spectral analysis for retinal perfusion	1–2 mm (OCTA)	Low (proof-of-concept)	High-resolution (e.g., retinal/septic changes); machine-learning integration	Feasibility studies only; standardization needed	5 (e.g., OCTA trial 2025; hyperspectral in sepsis 2024–2025)

* Approximate; varies by device/tissue. Recent studies include post-2025 updates (e.g., OCTA as “new frontier”).

## Data Availability

No new data were created or analyzed in this study.

## Abbreviations

The following abbreviations are used in this manuscript:

PPG	photoplethysmography
rPPG	remote photoplethysmography
aCRT	automated capillary refill time
CRT	capillary refill time
Q-CRT	Quantitative capillary refill time
PI	Perfusion index
NIRS	near-infrared spectroscopy
StO_2_	tissue oxygen saturation
VOT	vascular occlusion test
LSCI	laser speckle contrast imaging
SDF	sidestream dark-field imaging
IDF	incident dark-field imaging
FCD	functional capillary density
PPV	proportion of perfused vessels
MFI	microvascular flow index
PLR	passive leg rising
POCUS	point-of-care ultrasound
TTE	transthoracic echocardiography
MAP	mean arterial pressure

## References

[1] Singer M., Deutschman C.S., Seymour C.W., Shankar-Hari M., Annane D., Bauer M., Bellomo R., Bernard G.R., Chiche J.-D., Coopersmith C.M. (2016). The Third International Consensus Definitions for Sepsis and Septic Shock (Sepsis-3). JAMA.

[2] (2025). GBD 2021 Global Sepsis Collaborators. Global, regional, and national sepsis incidence and mortality, 1990–2021: A systematic analysis. Lancet Glob. Health.

[3] Ince C. (2015). Hemodynamic coherence and the rationale for monitoring the microcirculation. Crit. Care.

[4] De Backer D., Donadello K., Taccone F.S., Ospina-Tascon G., Salgado D., Vincent J.L. (2011). Microcirculatory alterations: Potential mechanisms and implications for therapy. Ann. Intensive Care.

[5] Hernandez G., Ospina-Tascon G.A., Damiani L.P., Estenssoro E., Dubin A., Hurtado J., Friedman G., Castro R., Alegria L., Teboul J.L. (2019). Effect of a Resuscitation Strategy Targeting Peripheral Perfusion Status vs Serum Lactate Levels on 28-Day Mortality Among Patients with Septic Shock: The ANDROMEDA-SHOCK Randomized Clinical Trial. JAMA.

[6] Bakker J., Nijsten M.W., Jansen T.C. (2013). Clinical use of lactate monitoring in critically ill patients. Ann. Intensive Care.

[7] Monnet X., Messina A., Greco M., Bakker J., Aissaoui N., Cecconi M., Coppalini G., De Backer D., Edul V.K., Evans L. (2025). ESICM guidelines on circulatory shock and hemodynamic monitoring 2025. Intensive Care Med..

[8] Haddaway N.R., Page M.J., Pritchard C.C., McGuinness L.A. (2022). PRISMA2020: An R package and Shiny app for producing PRISMA 2020-compliant flow diagrams, with interactivity for optimised digital transparency and Open Synthesis. Campbell Syst. Rev..

[9] De Backer D. (2023). Novelties in the evaluation of microcirculation in septic shock. J. Intensive Med..

[10] Bakker J., Ince C. (2020). Monitoring coherence between the macro and microcirculation in septic shock. Curr. Opin. Crit. Care.

[11] Sathianathan S., Sachar S., Berro J., Nero N., Bauer S.R., Tonelli A.R., Hamzaoui O., Siuba M.T. (2026). Vasoactive Medications and the Microcirculation in Septic Shock: A Scoping Review. Crit. Care Med..

[12] Dubin A. (2025). Effects of fluids on sublingual microcirculation: A point of view review. Ann. Intensive Care.

[13] Kanoore Edul V.S., Ince C., Estenssoro E., Ferrara G., Arzani Y., Salvatori C., Dubin A. (2015). The Effects of Arterial Hypertension and Age on the Sublingual Microcirculation of Healthy Volunteers and Outpatients with Cardiovascular Risk Factors. Microcirculation.

[14] Edul V.S., Enrico C., Laviolle B., Vazquez A.R., Ince C., Dubin A. (2012). Quantitative assessment of the microcirculation in healthy volunteers and in patients with septic shock. Crit. Care Med..

[15] Miranda M., Balarini M., Caixeta D., Bouskela E. (2016). Microcirculatory dysfunction in sepsis: Pathophysiology, clinical monitoring, and potential therapies. Am. J. Physiol.-Heart Circ. Physiol..

[16] Ostergaard L., Granfeldt A., Secher N., Tietze A., Iversen N.K., Jensen M.S., Andersen K.K., Nagenthiraja K., Gutierrez-Lizardi P., Mouridsen K. (2015). Microcirculatory dysfunction and tissue oxygenation in critical illness. Acta Anaesthesiol. Scand..

[17] De Backer D., Donadello K., Sakr Y., Ospina-Tascon G., Salgado D., Scolletta S., Vincent J.L. (2013). Microcirculatory alterations in patients with severe sepsis: Impact of time of assessment and relationship with outcome. Crit. Care Med..

[18] Donati A., Damiani E., Domizi R., Scorcella C., Carsetti A., Tondi S., Monaldi V., Adrario E., Romano R., Pelaia P. (2016). Near-infrared spectroscopy for assessing tissue oxygenation and microvascular reactivity in critically ill patients: A prospective observational study. Crit. Care.

[19] Sato J., Sakurai A., Ihara S., Nakagawa K., Chiba N., Saito T., Kinoshita K. (2024). Assessment of Microcirculatory Dysfunction by Measuring Subcutaneous Tissue Oxygen Saturation Using Near-Infrared Spectroscopy in Patients with Circulatory Failure. Diagnostics.

[20] Hendrick E., Jamieson A., Chiesa S.T., Hughes A.D., Jones S. (2024). A short review of application of near-infrared spectroscopy (NIRS) for the assessment of microvascular post-occlusive reactive hyperaemia (PORH) in skeletal muscle. Front. Physiol..

[21] Cody N., Bradbury I., McMullan R.R., Quinn G., O’Neill A., Ward K., McCann J., McAuley D.F., Silversides J.A. (2024). Physiologic Determinants of Near-Infrared Spectroscopy-Derived Cerebral and Tissue Oxygen Saturation Measurements in Critically Ill Patients. Crit. Care Explor..

[22] Menezes I.A.C., Cunha C., Carraro Junior H., Luy A.M. (2018). Perfusion index for assessing microvascular reactivity in septic shock after fluid resuscitation. Rev. Bras. Ter. Intensiv..

[23] Rasmy I., Mohamed H., Nabil N., Abdalah S., Hasanin A., Eladawy A., Ahmed M., Mukhtar A. (2015). Evaluation of Perfusion Index as a Predictor of Vasopressor Requirement in Patients with Severe Sepsis. Shock.

[24] Elshal M.M., Hasanin A.M., Mostafa M., Gamal R.M. (2021). Plethysmographic Peripheral Perfusion Index: Could It Be a New Vital Sign?. Front. Med..

[25] Er M.C., Kaya C., Ustun Y.B., Sahinoglu A.H. (2020). Predictive value of perfusion index for mortality in mechanically ventilated patients. Aging Male.

[26] Shi X., Xu M., Yu X., Lu Y. (2020). Peripheral perfusion index predicting prolonged ICU stay earlier and better than lactate in surgical patients: An observational study. BMC Anesthesiol..

[27] Lombardi S., Partanen P., Francia P., Calamai I., Deodati R., Luchini M., Spina R., Bocchi L. (2022). Classifying sepsis from photoplethysmography. Health Inf. Sci. Syst..

[28] Coutrot M., Dudoignon E., Joachim J., Gayat E., Vallee F., Depret F. (2021). Perfusion index: Physical principles, physiological meanings and clinical implications in anaesthesia and critical care. Anaesth. Crit. Care Pain Med..

[29] Sun X., He H., Xu M., Long Y. (2024). Peripheral perfusion index of pulse oximetry in adult patients: A narrative review. Eur. J. Med. Res..

[30] He H.W., Liu W.L., Zhou X., Long Y., Liu D.W. (2020). Effect of mean arterial pressure change by norepinephrine on peripheral perfusion index in septic shock patients after early resuscitation. Chin. Med. J..

[31] Ince C., Mayeux P.R., Nguyen T., Gomez H., Kellum J.A., Ospina-Tascon G.A., Hernandez G., Murray P., De Backer D., Workgroup A.X. (2016). The Endothelium in Sepsis. Shock.

[32] Schmidt E.P., Yang Y., Janssen W.J., Gandjeva A., Perez M.J., Barthel L., Zemans R.L., Bowman J.C., Koyanagi D.E., Yunt Z.X. (2012). The pulmonary endothelial glycocalyx regulates neutrophil adhesion and lung injury during experimental sepsis. Nat. Med..

[33] Iba T., Levy J.H., Wada H., Thachil J., Warkentin T.E., Levi M., Subcommittee on Disseminated Intravascular C. (2019). Differential diagnoses for sepsis-induced disseminated intravascular coagulation: Communication from the SSC of the ISTH. J. Thromb. Haemost..

[34] Tang A., Shi Y., Dong Q., Wang S., Ge Y., Wang C., Gong Z., Zhang W., Chen W. (2024). Prognostic Value of Sublingual Microcirculation in Sepsis: A Systematic Review and Meta-analysis. J. Intensive Care Med..

[35] Zhang X., Zhang H., Jin R., Li L., Huang L., Wang Z., Peng Q., Ai M., Zhang L. (2026). Assessment of Sublingual Microcirculation to Evaluate the Efficacy of Resuscitation Therapy in Septic Shock Patients: A cohort study. Shock.

[36] Hernandez G., Ospina-Tascon G.A., Kattan E., Ibarra-Estrada M., Ramasco F., Orozco N., ANDROMEDA-SHOCK-2 Investigators for the ANDROMEDA Research Network, Spanish Society of Anesthesiology, Reanimation and Pain Therapy (SEDAR), Latin American Intensive Care Network (LIVEN) (2025). Personalized Hemodynamic Resuscitation Targeting Capillary Refill Time in Early Septic Shock: The ANDROMEDA-SHOCK-2 Randomized Clinical Trial. JAMA.

[37] Castro R., Kattan E., Ferri G., Pairumani R., Valenzuela E.D., Alegria L., Oviedo V., Pavez N., Soto D., Vera M. (2020). Effects of capillary refill time-vs. lactate-targeted fluid resuscitation on regional, microcirculatory and hypoxia-related perfusion parameters in septic shock: A randomized controlled trial. Ann. Intensive Care.

[38] Kazune S., Caica A., Volceka K., Suba O., Rubins U., Grabovskis A. (2019). Relationship of mottling score, skin microcirculatory perfusion indices and biomarkers of endothelial dysfunction in patients with septic shock: An observational study. Crit. Care.

[39] Gutierrez-Zarate D., Rosas-Sanchez K., Zaragoza J.J. (2023). Clinical evaluation of peripheral tissue perfusion as a predictor of mortality in sepsis and septic shock in the intensive care unit: Systematic review and meta-analysis. Med. Intensiva (Engl. Ed.).

[40] Machado F.R., Semler M.W. (2025). Capillary Refill Time in Sepsis-Searching for the Holy Grail. JAMA.

[41] Ait-Oufella H., Bige N., Boelle P.Y., Pichereau C., Alves M., Bertinchamp R., Baudel J.L., Galbois A., Maury E., Guidet B. (2014). Capillary refill time exploration during septic shock. Intensive Care Med..

[42] Yasufumi O., Morimura N., Shirasawa A., Honzawa H., Oyama Y., Niida S., Abe T., Imaki S., Takeuchi I. (2019). Quantitative capillary refill time predicts sepsis in patients with suspected infection in the emergency department: An observational study. J. Intensive Care.

[43] Dumas G., Lavillegrand J.R., Joffre J., Bige N., de-Moura E.B., Baudel J.L., Chevret S., Guidet B., Maury E., Amorim F. (2019). Mottling score is a strong predictor of 14-day mortality in septic patients whatever vasopressor doses and other tissue perfusion parameters. Crit. Care.

[44] Huang H., Wu C., Shen Q., Fang Y., Xu H. (2022). Value of variation of end-tidal carbon dioxide for predicting fluid responsiveness during the passive leg raising test in patients with mechanical ventilation: A systematic review and meta-analysis. Crit. Care.

[45] Monnet X., Shi R., Teboul J.L. (2022). Prediction of fluid responsiveness. What’s new?. Ann. Intensive Care.

[46] Pottecher J., Deruddre S., Teboul J.L., Georger J.F., Laplace C., Benhamou D., Vicaut E., Duranteau J. (2010). Both passive leg raising and intravascular volume expansion improve sublingual microcirculatory perfusion in severe sepsis and septic shock patients. Intensive Care Med..

[47] Messina A., Collino F., Cecconi M. (2020). Fluid administration for acute circulatory dysfunction using basic monitoring. Ann. Transl. Med..

[48] van Gastel M., Stuijk S., de Haan G. (2016). Robust respiration detection from remote photoplethysmography. Biomed. Opt. Express.

[49] Hassanpour A., Yang B. (2025). Contactless Vital Sign Monitoring: A Review Towards Multi-Modal Multi-Task Approaches. Sensors.

[50] Klibus M., Serova V., Rubins U., Marcinkevics Z., Grabovskis A., Sabelnikovs O. (2026). Pulse Waveform Changes During Vasopressor Therapy Assessed Using Remote Photoplethysmography: A Case Series. J. Clin. Med..

[51] Morimura N., Takahashi K., Doi T., Ohnuki T., Sakamoto T., Uchida Y., Takahashi H., Fujita T., Ikeda H. (2015). A pilot study of quantitative capillary refill time to identify high blood lactate levels in critically ill patients. Emerg. Med. J..

[52] Shinozaki K., Saeki K., Jacobson L.S., Falotico J.M., Li T., Hirahara H., Horie K., Kobayashi N., Weisner S., Lampe J.W. (2021). Evaluation of accuracy of capillary refill index with pneumatic fingertip compression. J. Clin. Monit. Comput..

[53] Ruan Z., Li R., Dong W., Cui Z., Yang H., Ren R. (2022). Laser speckle contrast imaging to monitor microcirculation: An effective method to predict outcome in patients with sepsis and septic shock. Front. Bioeng. Biotechnol..

[54] Hsieh M.C., Hu J.J., Lin Y.R., Li S.Y., Hsieh P.Y., Shing Ching C.T., Liao L.D. (2024). Improving the early diagnosis and clinical outcomes of shock patients via laser speckle contrast imaging assessment of peripheral hemodynamics. iScience.

[55] Heeman W., Steenbergen W., van Dam G., Boerma E.C. (2019). Clinical applications of laser speckle contrast imaging: A review. J. Biomed. Opt..

[56] Aksu U., Yavuz-Aksu B., Goswami N. (2024). Microcirculation: Current Perspective in Diagnostics, Imaging, and Clinical Applications. J. Clin. Med..

[57] Aykut G., Veenstra G., Scorcella C., Ince C., Boerma C. (2015). Cytocam-IDF (incident dark field illumination) imaging for bedside monitoring of the microcirculation. Intensive Care Med. Exp..

[58] Dietrich M., Marx S., von der Forst M., Bruckner T., Schmitt F.C.F., Fiedler M.O., Nickel F., Studier-Fischer A., Muller-Stich B.P., Hackert T. (2021). Bedside hyperspectral imaging indicates a microcirculatory sepsis pattern—An observational study. Microvasc. Res..

